# Non-inflammatory pancreatic cysts: from diagnosis to treatment (97 cases series)

**DOI:** 10.1590/0100-6991e-20213009

**Published:** 2021-11-05

**Authors:** ENIO CAMPOS AMICO, CAIO TRAJANO SIQUEIRA SALGADO, JOSÉ ROBERTO ALVES, ADRIANO DE ARAÚJO LIMA LIGUORI, ROGÉRIO LACERDA SOUSA

**Affiliations:** 1 - Centro de Gastroenterologia e Endoscopia Digestiva de Natal, GASTROCENTRO - Natal - RN - Brasil; 2 - Universidade Federal do Rio Grande do Norte (UFRN), Departamento de Medicina Integrada - Natal - RN - Brasil; 3 - Universidade Federal de Santa Catarina, Departamento de Cirurgia - Florianopolis - SC - Brasil; 4 - Universidade Federal do Rio Grande do Norte (UFRN), Unidade de Diagnóstico por Imagem e Métodos Gráficos do Hospital Universitário Onofre Lopes - Natal - RN - Brasil

**Keywords:** Pancreatic Cyst, Diagnosis, Therapy, Pancreas, Digestive System Surgical Procedures, Cisto Pancreático, Diagnóstico, Terapêutica, Pâncreas, Procedimentos Cirúrgicos do Sistema Digestório

## Abstract

**Objective:**

to describe the implications of the diagnosis and treatment of non-inflammatory pancreatic cysts in a series of patients.

**Methods:**

we included patients with pancreatic cysts ≥1.0 cm, excluding those with a presumptive diagnosis of a pseudocyst. Imaging tests, echoendoscopy, and histopathology determined the diagnosis of the type of cyst. We applied the guidelines of the International Association of Pancreatology, with some modifications, in patients with mucinous or indeterminate lesions.

**Results:**

97 adult patients participated in the study. A cystic neoplasm of the pancreas was diagnosed in 82.5% of cases. Diagnosis was mainly made by magnetic resonance (46% of cases). The two most common diagnoses were intraductal papillary mucinous neoplasm (43.3%) and serous cystadenoma (26%). Twenty-nine patients underwent surgery (33.3%). The most common surgical procedure was distal pancreatectomy associated with splenectomy in 19 cases (65.5%). Among the operated patients, 11 were diagnosed with cancer. None of the followed, non-operated patients had a diagnosis of cancer.

**Conclusions:**

magnetic resonance showed good accuracy, particularly in the diagnosis of intraductal papillary mucinous neoplasm. The guidelines of the International Association of Pancreatology, as applied in this study, showed a negative predictive value for cancer of 100%. A development of better diagnostic tests can reduce the number of unnecessary operations.

## RESUMO

**Objetivo:**

descrever as implicações do diagnóstico e tratamento dos cistos não inflamatórios do pâncreas em série de pacientes.

**Metódos:**

foram incluídos pacientes com cisto de pâncreas ≥1,0cm excluindo aqueles com diagnóstico presuntivo de pseudocisto. Exames de imagem, ecoendoscopia e anatomia-patológica determinaram o diagnóstico do tipo de cisto. As diretrizes da Associação Internacional de Pancreatologia foram aplicadas, com algumas modificações, nos pacientes com lesões mucinosas ou indeterminadas.

**Resultados:**

noventa e sete pacientes adultos participaram do estudo. A neoplasia cística de pâncreas foi diagnosticada em 82,5% dos casos. O diagnóstico foi feito principalmente por ressonância magnética (46% dos casos). Os dois diagnósticos mais frequentes foram a neoplasia papilar intraductal mucinosa (43,3%), e o cistoadenoma seroso (26%). Vinte e nove pacientes foram submetidos a operação (33,3%). O procedimento cirúrgico mais comum foi a pancreatectomia corpo-caudal associada à esplenectomia em 19 casos (65,5%). Entre os pacientes operados, 11 tiveram o diagnóstico de câncer. Nenhum dos pacientes seguidos teve o diagnóstico de câncer.

**Conclusões:**

a ressonância magnética apresentou boa acurácia, particularmente no diagnóstico da neoplasia papilar intraductal mucinosa. As diretrizes da Associação Internacional de Pancreatologia da forma que foram aplicadas no presente estudo, mostraram valor preditivo negativo para o câncer de 100%. O desenvolvimento de estratégias diagnósticas com melhor acurácia podem reduzir o número de cirurgias desnecessárias.

## INTRODUCTION

The pancreatic cyst, an increasingly frequently finding in clinical practice, can cause anxiety in patients, in addition to overloading the use of the health system due to the need for imaging and endoscopic exams, and even the demand for surgical treatment^1^ ^,^ ^2^. The concern with the diagnosis of malignant or potentially malignant lesions is legitimate, since unlike the cystic lesions in the liver and kidney, in the pancreas mucinous lesions with potential for malignant transformation are more frequent^3^. Once a pseudocyst is ruled out, the management of pancreatic cysts remains challenging, since the diagnosis, based on imaging and endoscopic exams, is not completely reliable^4^ ^,^ ^5^. In addition, even today surgical treatment is still associated with significant morbidity and mortality^6^. When analyzing the Brazilian national scenario, there are only few studies on the subject, usually based on case reports^7^, already operated case series^8^ ^,^ ^9^, or case series submitted to some specific exam and no appropriate clinical or radiological follow-up^10^. The objective of this study was to evaluate a consecutive series of patients seeking the clinic of digestive tract diseases with pancreatic cyst, whose diagnosis of pseudocyst was ruled out and the one of pancreatic cystic neoplasia (PCN) was most likely. We studied the diagnostic and therapeutic challenges for these patients.

## METHOD

We evaluated patients treated at the Center for Gastroenterology and Digestive Endoscopy Gastrocentro in Natal and at the Onofre Lopes University Hospital (HUOL-UFRN), in Natal, State of Rio Grande do Norte, Brazil, between 2006 and 2020, with a diagnosis of pancreatic cyst with a size equal to or greater than 1.0 cm in diameter. We stratified patients into three groups: 1) typical PCN imaging computed tomography (CT), nuclear magnetic resonance (MRI), and echoendoscopy (EE) (with or without puncture and analysis of the aspirated fluid); 2) PCN confirmed in histopathological examination in cases undergoing surgical resection; and 3) nonspecific pancreatic cyst (NPC), when after the diagnostic investigation one could not establish the cyst’s etiology. We excluded patients who had alcohol (ethanol) ingestion exceeding 40 grams per day for more than five years, diagnosis of chronic pancreatitis, history of acute pancreatitis prior to the cyst diagnosis in the case of nonspecific injury, and follow-up time of less than one year for patients with NPC.

The imaging diagnosis of PCN was based on the classification by Sahani et al.^11^ and on the Fukuoka^12^ ^,^ ^13^ guidelines. At the time of patient inclusion, we also decided whether the treatment would be surgical or conservative. Surgical treatment of intraductal papillary mucinous neoplasm (IPMN) and of NPC was based on the guidelines of the International Association of Pancreatology (AIP), according to different publications: Sendai, 2006^14^ and Fukuoka, 2012^12^ and 2017^13^. We considered as “worrisome” factors, according to the Fukuoka (2012) guideline, a cyst size ≥3cm, thickening/enhancement of the cyst wall, main pancreatic duct (MPD) measuring between 5 and 9mm, mural nodule with no enhancement, and abrupt caliber change in the MPD with atrophy of the distal pancreas. The Fukuoka guidelines (2017) excluded the presence of mural nodule with no enhancement as a worrisome factor and included a mural nodule <5mm with enhancement, lymphadenopathy, high Ca 19-9, and growth of the cyst in ≥5mm in two years.

Some modifications were made in the guidelines: 1) The guidelines of Sendai, used between 2006 and 2012, indicated EE for all cysts from 1 to 3cm, which was not performed, since small cysts have small frequency of cancer and EE was not available between us during this period; 2) Some patients with two or more worrisome factors that had a low surgical risk agreed on undergoing operation without preoperative EE after the clarification of the risks and benefits; 3) Although EE has been performed in all patients for whom the initial evaluation indicated it according to the Fukuoka 2012^12^ and 2017^13^ guidelines, such an examination was not carried out in the follow-up of patients without worrisome factors whose indication stemmed only from the size of the cyst being between 2 and 3cm. For such cases, when EE could not be done, MRI replaced it.

Briefly, surgical indication generally occurred in the following situations: 1) presence of typical signs and symptoms related to the cyst; 2) diagnoses of mucinous cystadenoma and solid pseudopapillary tumor of the pancreas; 3) Diagnosis of main duct intraductal papillary mucinous neoplasm (MD-IPMN); 4) Patients with branch duct intraductal papillary mucinous neoplasm (BD-IPMN) or mixed intraductal papillary mucinous neoplasm (M-IPMN), when indicated by the guidelines; and 5) In those patients with BD-IPMN or M-IPMN with two or more worrisome factors. Surgery would take place after evaluation of the patient’s clinical condition, the type of pancreatectomy proposed, and the patient’s agreement after clarifying the procedure’s risks and benefits.

In the operated patients, the type of surgery was determined by the nature and location of the lesion. We categorized complications according to the Clavien-Dindo classification^15^ and defined pancreatic fistula and bleeding according to the International Study Group for Pancreatic Surgery^16^. Thus, Pancreatic fistula occured when the value of amylase in the drain’s liquid was greater than three times the serum amylase upper limit after the third postoperative day. Post-pancreatectomy bleeding was defined as loss of blood through the abdominal drain, abdominal cavity, or digestive tract, with a drop in levels of serum hemoglobin in the postoperative period. Postoperative mortality was death occurring within 90 days after operation.

For patients included in the follow-up group, imaging tests, preferably MRI, were repeated at variable time intervals according to the guidelines. Follow-up was defined individually for each case and kept until the age at which the patient maintained an acceptable surgical risk, usually between 80 and 85 years. This study was evaluated and approved by the Ethics in Research Committee of the Hospital Onofre Lopes, Federal University of Rio Grande do Norte (CAAE: 37529020.5.0000.5292).

## RESULTS

One hundred and fifty-two patients comprised the pancreatic cyst database. After applying the inclusion and exclusion criteria, we selected 97 patients to participate in the study. The mean age was 62.9 years (range 22-89), and the female sex was the most prevalent (83.5%). Most patients were asymptomatic (71.4%) and the symptomatic ones presented with nonspecific upper abdominal pain (25.5%) or with typical clinical signs of acute pancreatitis (4.1%). As for the number of lesions, in 71.1% of the patients the lesion was single, in 12.3% there were two or three lesions, and in 16.3%, four or more cysts. In patients with a single cyst, the distribution in the pancreatic parenchyma was: body/tail, 52.2%; head, 34.8%; neck, 7.2%; and uncinate process, 5.8% (Table 1).

**Table 1:** Baselines characteristics. diagnosis and treatment (n=97 patients).

Age	62.9 (22-89)
Gender (F:M)	81:16
Symptoms	
Asymptomatic	71.4%
Unspecific Symptoms	25.5%
Specific Symptoms	4.1%
Number of lesions	
Single	71,1%
2-3 lesions	12,3%
≥ 4 lesions	16,3%
Exam that defined the cyst diagnosis	
MRI	46%
CT	16%
MRI + EE	15%
Histopathology	15%
EE	8%
Definitive diagnosis	
Pancreatic Cystic Neoplasm	82.5%
Intraductal Papillary Mucinous Neoplasm	43.3%
Serous Cystadenoma	25.8%
Mucinous Cystadenoma	8.3%
Solid Pseudopapillary Tumor	4.1%
Neuroendocrine Tumor	1%
Nonspecific Pancreatic Cyst	17.5%
Definitive diagnosis of high-grade dysplasia/cancer	
Yes	11.3%
No	88.7%
Type of treatment	
Conservative treatment	70.1%
Immediate surgery	22 .7%
Surgery during follow-up	7.2%
Types of surgeries (n=29)	
Laparoscopic Distal Pancreatectomy + Splenectomy	34.5%
Open Distal Pancreatectomy + Splenectomy	31%
Pancreaticoduodenectomy	20.7%
Central Pancreatectomy	6.9%
Enucleation	3.4%
Exploratory Laparotomy	3.4%

**Figure 1:**
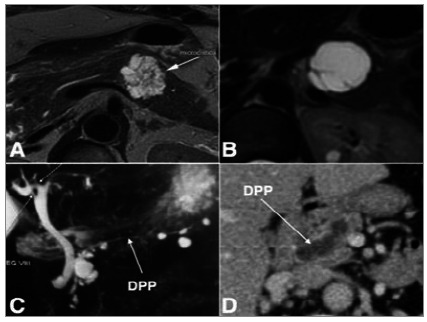
Typical PCN cases in imaging exams. **A.** MRI shows a microcystic lesion typical of SCA; **B.** MRI showing a pancreatic cyst with incomplete septa, typical of mucinous cystadenoma; **C. MRI with multiple BD-IPMN; the MPD with normal diameter is observed throughout its extension. D. CT showing a patient with MD-IPMN; there is dilated MPD (11mm) in the pancreatic body without an identifiable obstructive factor. PCN: pancreatic cystic neoplasm; MRI: magnetic resonance imaging; BD-IPMN: branch duct intraductal papillary mucinous neoplasm; SCA: serous cystadenoma; MPD: main pancreatic duct; CT: computerized tomography; MD-IPMN: main duct intraductal papillary mucinous neoplasm.
**

Eighty patients were diagnosed with PCN, while 17 were considered to have NPC. In patients with PCN, the diagnosis was made exclusively by MRI in 46% of cases, by CT in 16%, by histopathology in 15%, by the association of MRI and EE in 15%, and exclusively by EE in 8% of cases (Figure 1).

**Figure 2:**
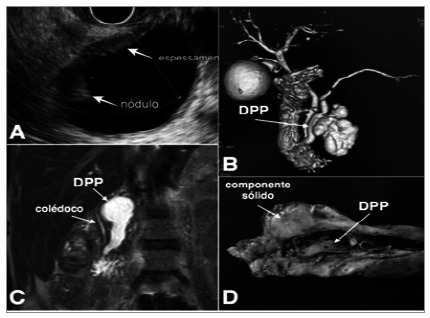
IPMN cases in endoscopic and imaging exams. **A. EE in a patient with BD-IPMN, in which a nodule is found on the cyst wall; B. MRI showing M-IPMN (7mm MPD); C. MRI showing MD-IPMN (20mm MPD); D. MD-IPMN surgical specimen showing a solid component (invasive adenocarcinoma). IPMN: intraductal papillary mucinous neoplasm; EE: echoendoscopy; BD-IPMN: branch duct intraductal papillary mucinous neoplasm; MRI: magnetic resonance imaging; M-IPMN: mixed intraductal papillary mucinous neoplasm; MD-IPMN: main duct intraductal papillary mucinous neoplasm; MPD: main pancreatic duct.
**

Twenty-two of the 97 patients underwent immediate operation (22.6%). Seventy-five patients were subjected to follow-up (77.3%). There was an average of 1.6 imaging exams per patient in the follow-up. Patients were followed through clinical and radiological evaluation for a mean time of 27.3 months (range 1-127). During follow-up, only seven out of 75 (9.3%) patients were operated on. The final diagnoses of the whole series were IPMN (42%), SCA (26%), NPC (17.5%), mucinous cystadenoma (8%), pseudopapillary solid of the pancreas (4%), and neuroendocrine tumor (1%) (Figure 2).

**Figure 3:**
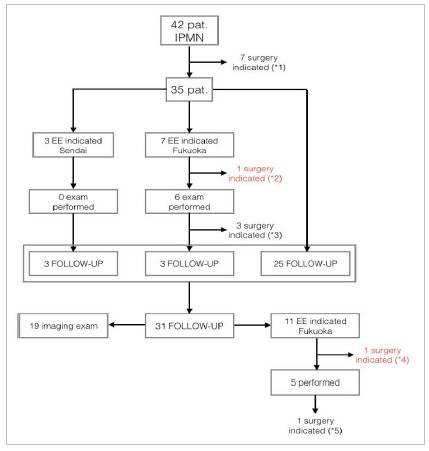
Flowchart of patients with IPMN with types of exams carried out, moment of surgical indication, and follow-up approach. In red, we highlight the cases of surgical indication outside the Fukuoka guideline. Reasons for surgical indication: (*1) five patients for suspected solid component and two for MPD >10mm; (*2) for >2 worrisome factors; (*3) two patients for suspected MPD involvement and one for solid component; (*4) for >2 worrisome factors; (*5) for suspected MPD involvement. IPMN: intraductal papillary mucinous neoplasm; MPD: main pancreatic duct.

Patients with IPMN were managed according to the guidelines of Sendai’s and Fukuoka 2012 and 2017 in four, 25, and 13 patients, respectively. Seven patients had immediate surgical indication after the initial imaging examination based on the algorithm (five for solid component; two for MPD ≥10mm). Seven patients had EE indication. Of these, in one case the operation was indicated without performing the exam (>2 worrisome factors) and the other six patients underwent the exam, of whom three had surgical indication after the exam (two due to involvement of the MPD and one due to a solid component). Of all patients with IPMN, 90.4% were followed up with at least one more imaging exam. The mean time of radiological follow-up in patients with IPMN was 25.2 months (range 1-117). Almost two thirds of the patients with IPMN (64.3%) were followed up with imaging exams for at least six months. In the follow-up, eleven patients had an indication for EE, one of them for the second time. Of the five patients who underwent EE, one had surgery indicated (suspected MPD involvement). The patient was operated on, and the diagnosis was BD-IPMN, without high-grade dysplasia or cancer. Nineteen patients were followed up only with imaging exams (CT or MRI). Figure 3 shows the algorithm followed to determine the surgical indication, type of exams performed, and follow-up of patients in the IPMN group.

Eight of the 13 patients with IPMN for whom surgery was indicated were operated. In four of the operated cases there was cancer (two BD-IPMN cases with adenocarcinoma; one BD-IPMN case with synchronous adenocarcinoma; and one MD-IPMN case with adenocarcinoma). Five patients were not operated, three of them due to advanced age (>80 years) associated with high surgical risk, one due to refusal, and one due to loss of follow-up.

In all five patients with IPMN and MPD involvement (three with MD-IPMN and two with M-IPMN) surgery was indicated. The reason for the indication in the three cases of MD-IPMN was the presence of an MPD greater than 10mm. In one of the M-IPMN cases, EE was performed and found papillary projections compatible with MPD involvement. In the other case of M-IPMN, the operation was indicated by the presence of two worrisome factors, (cyst larger than 3cm and MPD dilatation of 6mm). Only two of these five patients underwent operation. All patients who did not undergo surgery were alive with five, six, and eight years of follow-up.

In 17 patients the diagnosis was NPC. The mean age in this group was 64.2 years (range 46-89). The female:male ratio was 3.25:1. All patients were asymptomatic. The mean time of radiological follow-up was 27.5 months. Most cases corresponded to a single cyst (70.6%), the average size of the largest lesion being 1.7cm and generally located in the body-tail of the pancreas (47.2%). Multiple cysts across the entire length of the MPD were found in 17.6% of cases. In 41.2% there was an increase in the lesion during follow-up. EE was indicated in two patients (11.7%) and in one case there was indication for surgical treatment due to the diagnosis of a nodule in the cyst wall (solid component). The patient refused the procedure. 

In total, 29 patients underwent surgical treatment (33.3%). The three most frequent diagnoses were SCA (34.5%), IPMN (24.1%), and mucinous cystadenoma (24.1%). The most suitable type of surgical procedure was distal pancreatectomy associated with splenectomy in 19 cases (65.5%), 10 of which through laparoscopic access. Pancreaticoduodenectomy was performed in six cases (20.7%), central pancreatectomy in two cases (6.9%), enucleation in one case (3.4%), exploratory laparotomy in one patient with mucinous cystadenoma with cancer and peritoneal carcinomatosis (3.4%). Of the patients undergoing pancreatectomy (n=28), the Clavien-Dindo complication rate was 33.3%, 14.8%, 48.1%, and 3.7% for grades 0, I, II, and IIIA, respectively. Grade B and C fistula rate was 29.6%. The median drain time was nine days. Mortality was nil.

Based on clinical presentation, imaging exams, and histopathological findings, we identified 16 patients (55.2%) who would benefit from the surgical procedure, comprising seven cases of mucinous cystadenoma, four cases of pseudopapillary solid tumor, two cases of BD-IPMN with adenocarcinoma, one case of MD-IPMN with adenocarcinoma, one case of neuroendocrine tumor, and one case of adenocarcinoma. On the other hand, 13 patients (44.8%) were operated for injuries that could have been clinically followed. Of these, 10 were patients with SCA who had other preoperative diagnoses, and three with diagnosed MPD involvement (one by MRI and two by EE) não confirmados ao exame anatomo-patológico who had BD-IPMN without dysplasia or infiltrative cancer at histopathology.

Among the operated patients, 11 were diagnosed with cancer, all in the immediate operation group. The most common was pseudopapillary solid tumor (four cases), followed by BD-IPMN (two cases), mucinous cystadenoma (two cases), MD-IPMN (one case), BD-IPMN with the synchronous adenocarcinoma (one case), and neuroendocrine tumor (one case). Considering the whole sample, the incidence of cancer in MD-IPMN cases was 33.3%, 25% in mucinous cystadenoma, and in cases of BD-IPMN, 8.3%.

## DISCUSSION

Patients with non-inflammatory pancreatic cysts are increasingly frequent in gastroenterology clinics. The present study aimed to show the implications of the diagnosis and treatment of these patients from a “real life” perspective in the office. In general, in our series the non-inflammatory pancreatic cyst occurred more frequently as a single lesion in the body/tail of the pancreas, in asymptomatic women, in their sixth decade of life. Despite extensive investigation, in 17.5% of patients a specific diagnosis could not be reached. Although 22.6% of patients underwent immediate surgical treatment, clinical / radiological monitoring was the most common and proved to be safe, since 7.2% of patients needed later operation, and in none dysplasia or high-grade cancer was found.

In almost half of the patients, the diagnosis was made exclusively by MRI, which is considered the method of choice for identifying the communication between the cyst and the pancreatic ductal system, which is essential for the diagnosis of IPMN. In addition, MRI has the additional benefit of not emitting ionizing radiation^17^. Some authors, however, have shown that the diagnostic correlation between the nature of the pancreatic cyst and imaging exams is not perfect. Mohamed. et al.^18^, in a systematic review involving 22 publications, recognized that MRI specificity rates in this scenario are low (50 86%). We observed high specificity of MRI for IPMN, but low specificity for SCA. Del Chiaro et al^19^ retrospectively evaluated 141 patients subjected to surgical treatment by PCN and found an agreement between imaging and histopathological examination in patients with SCA in only 24.2% of cases. From our perspective, however, in this series, instead of MRI low accuracy, it is likely that the large number of SCA with atypical morphology (unilocular, bilocular, macrocystic, and solid) was responsible for this low specificity.

Some authors have suggested that EE may be useful as a routine test for diagnosing the nature of the pancreatic cyst. The dosage of the carcinoembryonic antigen in the cyst fluid has been used for differential diagnosis of mucinous and non-mucinous lesions^20^. The large variability between the cut-off values and the need to aspirate a volume of at least 1mL are limitations^21^. We could not define the usefulness of the carcinoembryonic antigen in the diagnosis of SCA in the present study, since only 36% of the cases of SCA were subjected to puncture and in only 12% of the cases the carcinoembryonic antigen was measured. In an electronic survey published by Westerveld D. et al.^22^ with members of the American Society for Gastrointestinal Endoscopy, half of the participating services requested EE for patients with an incidental pancreatic cyst smaller than 2cm. Besides there being no evidence of greater accuracy with EE, the study showed the higher costs and potential risks of the exam, as it is more invasive than the conventional imaging tests. Other strategies to improve the diagnosis of pancreatic cysts have been described, such as the search for genetic mutations and the immunohistocytochemical study, whether of the aspirated fluid^23^ ^,^ ^24^, or even more recently, of the microbiopsy of the cyst wall^25^, neither available in our environment.

Several guidelines have been published in the last 15 years to define, especially in cases of IPMN, which patients should be operated or only followed^12^ ^-^ ^14^. The guidelines applied to our patients were those published by the AIP, since they were the first proposed guidelines^14^ and have demonstrated a good negative predictive factor for cancer^26^. The modifications made in the study were adopted to reduce the number of performed EE, which, in our view, did not affect the guideline safety, since all those patients with worrisome factors were submitted to the exam. EE, according to our protocol, was indicated in one fourth of the IPMN cases, and in those patients in whom it was performed, it mostly (70%) endorsed conservative treatment. Due to the small number of exams carried out, we could not reach conclusions about the efficiency of EE. If, on the one hand, the exam was able to find a solid component in two cases that had not been seen on the imaging exam, on the other, it was not able to precisely define the involvement of the MPD. In two of the four patients that the EE identified such a commitment, this was not confirmed by the histopathologic exam.

Although with a small number of patients, this study demonstrated the safety of IPMN management with the applied protocolos protocol, since none of the patients followed for an average time of 25.2 months developed cancer. The efficiency of the Fukuoka guideline has recently been compared to the one from the American Gastroenterological Association in a meta-analysis by Wu et al.^27^, which included 21 studies and 3,723 patients. Although the authors found similar efficacy across the guidelines, the sensitivity for diagnosing cancer was greater with the Fukuoka.

Few patients with IPMN in the series had proven involvement of the MPD (five). Despite this, since three of these patients (two with MD-IPMN and one with M-IPMN) have not been subjected to the indicated operation, it was possible to follow each of them up for at least five years and follow the natural evolution. Interestingly, although one patient presented with recurrent pancreatitis, none of them developed cancer until the last examination. This alerted us to the fact that, in elderly patients, in whom the surgical risk is known to be greater, even the involvement of the MPD may not represent an absolute surgical indication. An interesting retrospective, multicenter study published by Crippa et al.^28^ evaluated 281 patients in high-risk and with worrisome factors according to the Fukuoka guideline, through follow-up with a median of 51 months. Although 68 (46%) of the 122 patients who had MD-IPMN or M-IPMN had progressed in aspects such as cyst size, MPD dilation, appearance of a mural nodule, or development of symptoms, the disease’s overall and specific survival for these patients was respectively 74.1% and 81.2%. These data disagree with previous publications reporting a high rate of cancer in patients with MD-IPMN, around 61.6% (36-100%). It is important to emphasize that the authors who reported a high rate of cancer studied mostly series of surgically resected cases^12^, which certainly overestimates the real incidence of cancer caused by the disease, as it only includes the most advanced cases.

In patients with BD-IPMN, the incidence of cancer was 8.3%. In one case the surgical specimen revealed a synchronous adenocarcinoma unrelated to the cystic lesion. Yamaguchi et al.^29^ were the first to report (2002) an association between IPMN and pancreatic synchronic adenocarcinoma unrelated to IPMN. The authors found such an association in 9.2% of the surgical specimens of patients operated on by IPMN, suggesting for the first time in the literature a universal defect of the pancreatic ductal system that would transform the epithelium into a premalignant condition. A more recent study from the University of Tokyo included 1,404 patients with BD-IPMN followed for an average of six years. Sixty-eight of these patients developed adenocarcinoma during follow-up, and 30 were synchronous to IPMN. Overall, the incidence of cancer in the series was 3.3%, 6.6%, and 15% at 5, 10, and 15 years, respectively^30^. These studies make clear the importance of not interrupting the follow-up of these patients and of the study of the entire organ at the imaging exam.

## CONCLUSION

Non-inflammatory pancreatic cysts are more frequently IPMN and SCA. Many of these cysts persist without specific diagnosis. The most useful test for diagnosis is MRI. While the diagnosis of IPMN is typically done with MRI, SCA was atypical in the imaging tests in a considerable number of the patients in our series. The AIP guidelines, as applied in this study, had a negative predictive factor for cancer of 100% for IPMN and NPC. The development of diagnostic alternatives with better accuracy can reduce the number of unnecessary operations, which in our study was high (44.8%).
